# Association Between a State Law Allowing Pharmacists to Dispense Naloxone Without a Prescription and Naloxone Dispensing Rates

**DOI:** 10.1001/jamanetworkopen.2019.20310

**Published:** 2020-01-31

**Authors:** Neha S. Gangal, Ana L. Hincapie, Roman Jandarov, Stacey M. Frede, Jill M. Boone, Neil J. MacKinnon, Kathleen Koechlin, Jolene DeFiore-Hyrmer, Amy Holthusen, Pamela C. Heaton

**Affiliations:** 1 Department of Pharmacy Practice and Administrative Sciences, Winkle College of Pharmacy, University of Cincinnati, Cincinnati, Ohio; 2 Department of Environmental Health, University of Cincinnati, Cincinnati, Ohio; 3 Clinical Program Development, Kroger Health, Cincinnati, Ohio; 4 Violence & Injury Prevention Section, Ohio Department of Health, Columbus; 5 Project DAWN, Violence & Injury Prevention Section, Ohio Department of Health, Columbus (at time of study); 6 Division of Pharmacy Practice and Administrative Sciences, Winkle College of Pharmacy, University of Cincinnati, Cincinnati, Ohio

## Abstract

**Importance:**

Between 2015 and 2017, Ohio had the second highest number of opioid-related deaths. In July 2015, the Ohio General Assembly approved a law allowing pharmacists to dispense naloxone without a prescription in accordance with a physician-approved protocol. This change in the law allowed pharmacists to have more opportunity to participate in the management of patients who were addicted to opioids.

**Objective:**

To determine the association between the implementation of an Ohio law allowing pharmacists to dispense naloxone without a prescription in accordance with a physician-approved protocol and naloxone dispensing rates.

**Design, Setting, and Participants:**

A segmented regression analysis of an interrupted time series was performed for 30 consecutive months to evaluate the change in the naloxone dispensing rate before and after the implementation of the state law. Ohio Medicaid naloxone claims and Kroger Pharmacy naloxone claims for all 88 counties in Ohio were examined. Any patient 18 years or older with at least 1 naloxone order dispensed through Ohio Medicaid or by a Kroger Pharmacy in Ohio during the study period of July 16, 2014, to January 15, 2017, was included in the study. Data were analyzed from April 23, 2018, to July 7, 2019.

**Exposures:**

The primary independent variable was implementation of an Ohio law allowing pharmacists to dispense naloxone without a prescription in accordance with a physician-approved protocol, which took effect in July 2015.

**Main Outcomes and Measures:**

The primary outcome measure was the naloxone dispensing rate per month per county.

**Results:**

In the Ohio Medicaid population, the number of naloxone orders dispensed after the policy was implemented increased by 2328%, from 191 in the prepolicy period to 4637 in the postpolicy period. The rate of naloxone orders dispensed per month per county after the policy was implemented increased by 4% in the Ohio Medicaid population and 3% in the Kroger Pharmacy population compared with the prepolicy period. The rate of naloxone orders dispensed after the policy was implemented increased by 18% per month in low-employment counties compared with high-employment counties in the Ohio Medicaid population.

**Conclusions and Relevance:**

The implementation of a state law allowing pharmacists to dispense naloxone without a prescription in accordance with a physician-approved protocol was associated with an increase in the number of naloxone orders dispensed in the Ohio Medicaid and Kroger Pharmacy populations. Moreover, a significant increase was observed in the naloxone dispensing rate among the Ohio Medicaid population in counties with low employment and high poverty.

## Key Points

**Question:**

Is an Ohio law allowing pharmacists to dispense naloxone without a prescription in accordance with a physician-approved protocol associated with an increase in naloxone dispensing rates?

**Findings:**

In this cohort study of patients in Ohio who received naloxone, the number of naloxone orders dispensed after the state law was implemented increased by 2328%, and the naloxone dispensing rate per month per county increased significantly in the Ohio Medicaid (4%) and Kroger Pharmacy (3%) populations. In the Ohio Medicaid population, the naloxone dispensing rate in low-employment counties increased by 18% compared with high-employment counties.

**Meaning:**

The implementation of an Ohio law allowing pharmacists to dispense naloxone without a prescription was associated with a significant increase in naloxone dispensing rates, especially in low-employment counties.

## Teaser

This cohort study uses Ohio Medicaid and Kroger Pharmacy claims data and Area Health Resource File data to examine the association between a 2015 Ohio law allowing pharmacists to dispense naloxone without a prescription in accordance with a physician-approved protocol and naloxone dispensing rates.

## Introduction

Opioid misuse has substantial consequences for individuals and society. Deaths associated with drug overdoses have doubled in the last decade, with 70 200 overdose-related deaths reported in the United States in 2017.^1^ In 2016, 40% of the opioid-related overdose deaths were associated with the use of prescription opioids.^2^ Ohio has been particularly devastated by the opioid crisis. Between 2015 and 2017, Ohio had the second highest number of opioid-related deaths among all states.^3,4^ The death rate related to unintentional drug poisonings in Ohio, which is largely associated with opioid-related overdose, increased by 1081% between 2000 and 2017.^5^

Naloxone, a noncontrolled prescription medication, is used to reverse an opioid overdose. The Network for Public Health Law has stated that the laws concerning access to naloxone present both a problem and a solution.^6^ The problem is that laws regulating the use of prescription medications may limit access. To prevent death, naloxone must be readily available within minutes of an overdose; however, the occurrence of an overdose event is difficult to predict. A layperson may successfully administer naloxone,^7^ but the person must have access to the medication. The solution is to change laws to expand access to naloxone.

Because of the efforts of many organizations, such as the American Public Health Association, the National Association of Boards of Pharmacy, the US Conference of Mayors, and the American Medical Association, all states have increased access to naloxone by removing some legal barriers.^6^ In July 2015, the Ohio 131st General Assembly passed Ohio House Bill 4 (HB 4),^8^ which further expanded access to naloxone by permitting pharmacists, among others, to dispense naloxone without a prescription in accordance with a physician-approved protocol (also referred to as a standing order).^9^ Per this law, naloxone may be dispensed to an individual when there is a reason to believe the individual is experiencing or at risk of experiencing an opioid-related overdose, and it may be dispensed to a family member, friend, or other person in a position to assist an individual when there is a reason to believe the individual is at risk of experiencing an opioid-related overdose.^9^ Approximately 75% of community pharmacies in Ohio were registered to dispense naloxone without a prescription as of May 2019.^10^

Some evidence exists that reducing legal barriers may increase access to naloxone. A 2017 study by Rees et al^11^ reported that in states that implemented laws to expand access to naloxone, a 9% to 11% reduction occurred in opioid-related deaths. These findings were supported by a similar study by McClellan et al,^12^ which found that states that implemented naloxone access laws had a 14% lower incidence of deaths related to opioid overdose compared with states that did not have laws that increased naloxone access. Moreover, a study by Gertner et al^13^ reported that the presence of any naloxone access-related law increased the number of naloxone orders dispensed in the Medicaid population; the presence of a standing-order policy had a major role in this increase. However, substantiation of the findings by Gertner et al^13^ is needed, and evidence to support the benefit of a physician-approved protocol for patients who are not covered by Medicaid programs is lacking. Therefore, the objective of this study was to assess the association between the implementation of an Ohio law that allowed pharmacists to provide naloxone without a prescription in accordance with a physician-approved protocol and naloxone dispensing rates. This study enhances the evidence base to inform policy makers regarding whether dispensing naloxone according to a physician-approved protocol is associated with increases in access to naloxone.

## Methods

### Data Source, Design, and Population

This retrospective cohort study used Ohio Medicaid claims data, Kroger Pharmacy claims data for Ohio, and Area Health Resource File data. Ohio Medicaid and Kroger Pharmacy data contained patient and medication-dispensing history. Kroger Pharmacy data are an all-payer data source, providing information regarding patients who pay for medications with cash and patients who have third-party health insurance plans. Kroger Pharmacy is a large community pharmacy chain that operates more than 2300 pharmacies across 35 states. In response to Ohio HB 4, Kroger Pharmacy implemented a physician-approved protocol for naloxone in their 199 pharmacy locations in Ohio. The Area Health Resource File is a health resource database that contains information on health care professions, health facilities, population, economics, hospital use, and expenditures at the county, state, and national levels from more than 50 data sources.^14^

The study sample included all patients 18 years and older with at least 1 naloxone order dispensed through Ohio Medicaid or by a Kroger Pharmacy during the study period of July 16, 2014, to January 15, 2017. The naloxone dispensing rate per month was measured for 1 year before (prepolicy) and 18 months after (postpolicy) the passage of Ohio HB 4. This study followed the Strengthening the Reporting of Observational Studies in Epidemiology (STROBE) reporting guideline for cohort studies and was approved by the institutional review board of the University of Cincinnati with a waiver of consent, as all data were deidentified and examined retrospectively.

### Outcome Measures

The primary outcome measure was the rate of naloxone orders dispensed. For Ohio Medicaid data, the naloxone dispensing rate was calculated as the number of naloxone orders dispensed per month per county divided by the number of Medicaid beneficiaries for that county. For Kroger Pharmacy data, the naloxone dispensing rate was calculated as the number of naloxone orders dispensed per month per county divided by the county population. No indicator on the claims data was available to specifically designate that an order was dispensed in accordance with a physician-approved protocol, so we developed a proxy measure that we called physician proxy.

For the Ohio Medicaid subgroup analysis, we determined whether a naloxone order was dispensed via the physician-proxy measure by contacting the 3 large pharmacy chains that have the largest market share in Ohio, and we obtained the name of the physician authorizing their protocol. Using the physician’s National Provider Identifier, which is a unique 10-digit identification number issued to health care professionals by the Centers for Medicare and Medicaid Services, we identified naloxone orders dispensed under their names. The dispensing rate for this subgroup analysis was calculated as the number of physician-proxy naloxone orders divided by the number of naloxone orders dispensed by the 3 large chain community pharmacies. We performed a similar analysis with the Kroger Pharmacy claims data; however, we only used the National Provider Identifier of the physician specific to the Kroger protocol.

### Independent Variables

The primary independent variable was binary and indicated the implementation of Ohio HB 4, which allowed the physician-approved protocol. For the descriptive analysis, naloxone order–level and patient-level information, such as age, sex, and race, were obtained directly from the claims data. For the regression model, covariates described the demographic characteristics of the county, which included sex, race (white, black, and other), median age, median household income, unemployment rate, educational level, and county classification (low-education, low-employment, high-poverty, or health professional shortage area). Binary variables were created using data from the Area Health Resource File.

Season was included as a categorical variable with 4 categories—summer, autumn, winter, and spring—and was incorporated into the models to examine whether seasonality was associated with the naloxone dispensing rate.

### Data Analysis

The Ohio Medicaid and Kroger Pharmacy data were analyzed separately to reduce the risk of overestimation. County-level variables were added to each data set using information from the Area Health Resource File. The Kroger Pharmacy claims data were used to perform a confirmatory analysis of the Ohio Medicaid analysis. The descriptive analysis included frequencies and percent changes for naloxone order–level and patient-level measures for the Ohio Medicaid population. A segmented regression analysis, which controls for changes in the underlying trend of the outcome, was used to determine the longitudinal effect of the intervention.^15,16,17^ A regression analysis was performed for 30 consecutive months to evaluate the change in the naloxone dispensing rate before and after the implementation of the policy. For each month, the rate of naloxone claims per county was calculated. One interruption was introduced during the study period on the day that Ohio HB 4 went into effect on July 16, 2015. The postpolicy study period was 18 months compared with the prepolicy study period of 12 months; we controlled for this difference in length in the regression model by measuring the dependent variable as a rate. Data were analyzed from April 23, 2018, to July 7, 2019.

We had 4 regression models, 2 for the Ohio Medicaid data and 2 for the Kroger Pharmacy data that were used for the confirmatory analysis. For each data set, we had a regression model for each of the dependent variables; the first model included the overall dispensing rate, and the second model included the physician-proxy dispensing rate. The Durbin-Watson statistic, used to check for the presence of autocorrelation, was close to 2.00, indicating that no autocorrelation was present, which was expected because the rate was calculated as the percentage of the total county population and not as the increase in the number of naloxone orders dispensed compared with the previous month.^15^ We performed additional sensitivity analyses to confirm the linearity of the function by adding a second-degree polynomial of time variables; these time variables were not statistically significant, while the linear component of our model remained significant.

## Results

In the Ohio Medicaid population, the number of naloxone orders dispensed in the postpolicy period increased by 2328%, from 191 in the prepolicy period to 4637 in the postpolicy period (Table 1). The number of orders dispensed by the 3 large chain community pharmacies increased by 3237%, from 59 in the prepolicy period to 1969 in the postpolicy period. The most commonly used naloxone delivery methods were intramuscular, which may have been used intranasally with an atomizer (ie, off-label), and nasal (ie, Narcan, which was approved by the US Food and Drug Administration on November 18, 2015). The Figure presents a time series of the monthly number of naloxone orders dispensed in the Ohio Medicaid population before and after Ohio HB 4 was approved, and it depicts the increase in the number of naloxone orders dispensed in the postpolicy period. The total number of patients receiving naloxone increased 2000%, from 183 in the prepolicy period to 3847 in the postpolicy period (Table 2). No differences in sex, race, or geographic location were observed among patients in the prepolicy vs postpolicy period. However, patients residing in a low-employment county were significantly more likely to receive naloxone in the postpolicy period (11.5%) compared with the prepolicy period (3.8%; *P* = .001). Patients residing in a high-poverty county were also significantly more likely to receive naloxone in the postpolicy period (13.6%) compared with the prepolicy period (6.0%; *P* = .003). In the Kroger Pharmacy population, 50% of patients paid for their medication with cash or were covered by Medicare or private third-party health insurance plans.

**Table 1.** Naloxone Order–Level Measures in Ohio Medicaid Population

Measure	Prepolicy, No. (%)	Postpolicy, No. (%)
Naloxone orders, % increase	191	4637 (2327.8)
Orders per store, mean (SD)^a^	0.25 (1.48)	6.09 (28.33)
Naloxone orders dispensed by 3 largest community pharmacy chains	59 (30.9)	1969 (42.5)
Naloxone orders dispensed according to physician-approved protocol by 3 largest community pharmacy chains	0	767 (39.0)
Naloxone orders dispensed per drug delivery method		
Intramuscular		
Naloxone hydrochloride 0.4 mg/mL	21 (11.0)	127 (2.7)
Naloxone hydrochloride 1 mg/mL	143 (74.9)	1959 (42.2)
Nasal		
Naloxone hydrochloride 4 mg/actuation	0	2230 (48.1)
Autoinjector		
Naloxone HCl 0.4 mg/0.4 mL	27 (14.1)	321 (6.9)

^a^ Mean orders per store were calculated using the total number of stores that dispensed naloxone, not the total stores in Ohio, as the denominator.

**Figure.**
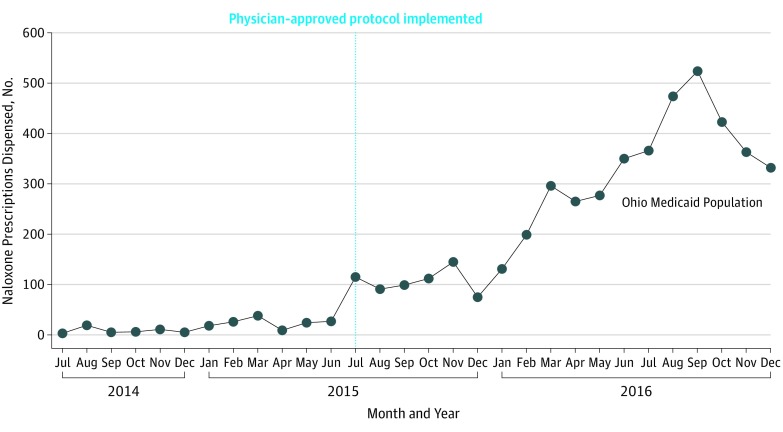
Number of Naloxone Orders Dispensed Among Ohio Medicaid Population Before and After Policy Implementation, by Month

**Table 2.** Patient-Level Measures in Ohio Medicaid Population

Measure	Prepolicy, No. (%) (n = 183)	Postpolicy, No. (%) (n = 3847)	*P* Value
Naloxone orders per person, mean (SD)	0.05 (0.22)	1.16 (0.61)	<.001
Age, mean (SD)	35 (12.49)	35 (10.96)	.99
Age range, y			.19
18-20	12 (6.55)	146 (3.80)	
21-30	67 (36.61)	1359 (35.33)	
31-40	57 (31.15)	1272 (33.06)	
41-50	25 (13.66)	623 (16.19)	
51-64	18 (9.84)	412 (10.71)	
≥65	4 (2.19)	35 (0.91)	
Sex			.13
Male	67 (36.61)	1628 (42.32)	
Female	116 (63.39)	2219 (57.68)	
Race			.74
White	149 (81.42)	3047 (79.20)	
Black	11 (6.01)	278 (7.23)	
Other	23 (12.57)	522 (13.57)	
Geography			.08
Metropolitan	158 (86.34)	3121 (81.13)	
Nonmetropolitan	25 (13.66)	726 (18.87)	
Low-employment county^a^			.001
Yes	7 (3.83)	441 (11.46)	
No	176 (96.17)	3406 (88.54)	
High-poverty county^a^			.003
Yes	11 (6.01)	522 (13.57)	
No	172 (93.99)	3325 (86.43)	

^a^ Includes patients who are living in a county classified as low employment or high poverty by the Area Health Resource File and does not indicate that patients are unemployed or belong to a lower socioeconomic class.

Table 3 presents the results of the 2 segmented regression analyses for the Ohio Medicaid population. For both the overall dispensing rate and the physician-proxy models, the baseline results indicated that before the implementation of Ohio HB 4, no significant month-to-month change occurred in the rate of naloxone dispensed. In addition, for both models, the postpolicy trend change, which reflected the change in the trend of the month-to-month naloxone dispensing rate after the policy took effect compared with before the policy took effect, indicated that the rate of naloxone dispensed in the postpolicy period increased significantly and compounded over time. In the overall model, the rate of naloxone dispensed after the policy was implemented increased by 4% per month per county, and in the physician-proxy model, the dispensing rate increased by 8% per month per county. The postpolicy level change reflected the immediate change in the rate of naloxone dispensed at the time of policy implementation. For the overall model only, an immediate increase of 17% was observed in the rate of naloxone dispensed after the policy change, indicating no lag period. In addition, we found that the rate of naloxone dispensed in low-employment counties compared with high-employment counties increased by 18% per month in the postpolicy period.

**Table 3.** Naloxone Dispensing Rate in Ohio Medicaid Population After Policy Change

Variable	Overall		Physician Proxy^a^	
	Risk Ratio (SE)	*P* Value	Risk Ratio (SE)	*P* Value
Intercept	0.01 (0.51)	<.001	0.019 (1.42)	.01
Baseline (β_1_)^b^	1.00 (0.01)	.61	010 (0.02)	.89
Postpolicy level change (β_2_)^c^	1.17 (0.05)	.002	1.07 (0.16)	.68
Postpolicy trend change (β_3_)^d^	1.04 (0.01)	<.001	1.08 (0.02)	.002
Season				
Autumn	1.01 (0.03)	.66	1.06 (0.08)	.49
Spring	1.02 (0.03)	.57	1.37 (0.1)	.002
Winter	0.94 (0.03)	.04	1.02 (0.1)	.85
Summer (Ref)				
Metropolitan area	1.06 (0.05)	.21	1.21 (0.13)	.15
County				
Low education	1.20 (0.16)	.27	0.99 (0.46)	.99
Low employment	1.18 (0.07)	.02	0.99 (0.20)	.98
High poverty	1.06 (0.1)	.51	1.21 (0.27)	.47
Health professional shortage area				
Mental health	1.09 (0.06)	.13	0.99 (0.16)	.94
Primary care	1.05 (0.04)	.32	1.17 (0.12)	.20
Median age	1.02 (0.01)	.14	0.98 (0.03)	.57
Sex^e^				
Male	1.05 (0.04)	.86	0.91 (0.10)	.37
Female	1.03 (0.04)	.46	0.91 (0.12)	.42
Race^e^				
White	1.0 (0.04)	.78	1.12 (0.10)	.27
Black	1.0 (0.04)	.96	1.13 (0.11)	.26
Other	1.0 (0.05)	.44	0.92 (0.12)	.52
Median household income^e^	1.0 (0.004)	.46	0.97 (0.01)	.03
Education^e^				
No high school diploma	1.0 (0.02)	.91	1.23 (0.06)	<.001
High school diploma	0.98 (0.08)	.01	0.94 (0.02)	.002
Some college or more	1.02 (0.01)	.03	1.11 (0.02)	<.001

^a^ Physician proxy indicates the rate of naloxone orders dispensed by physicians who could authorize the physician-approved protocol for 3 large pharmacy chains in Ohio.

^b^ The coefficient β_1_ is the effect of the continuous time variable, beginning with a value of 1 on July 16, 2014, and increasing by 1 each month. This coefficient represents a monthly time series.

^c^ The coefficient β_2_ is the effect of a binary indicator representing policy implementation on July 16, 2015, with a value of 0 before July 16, 2015, and 1 after July 16, 2015. This coefficient represents the immediate change in rate of naloxone orders after policy implementation.

^d^ The coefficient β_3_ is the effect of a continuous variable measuring the time passed after the implementation of the policy, beginning with a value of 0 and increasing by 1 each month; non-0 values began on August 16, 2015, 1 month after the policy was implemented.

^e^ Continuous variables depicting the total number of respective populations in the state per year. For these variables, the risk ratio reflects the change per 1000 increase.

Similar to the Ohio Medicaid analyses, the confirmatory analyses using Kroger Pharmacy data had 2 separate regression models for each of the dependent variables (Table 4). The baseline results indicated no significant month-to-month change in the rate of naloxone dispensed. The rate of naloxone dispensed per month per county, designated by the postpolicy trend change, increased significantly by 3% in the overall model and by 17% in the physician-proxy model during the postpolicy period. These results from the multipayer database are consistent with the Ohio Medicaid findings.

**Table 4.** Confirmatory Analysis of Naloxone Dispensing Rate After Policy Change^**a**^

Variable	Overall		Physician Proxy^b^	
	Risk Ratio (SE)	*P* Value	Risk Ratio (SE)	*P* Value
Intercept	0.002 (0.61)	<.001	0.001 (2.70)	.01
Baseline (β_1_)^c^	1.00 (0.05)	.82	1.00 (0.03)	.93
Postpolicy level change (β_2_)^d^	1.01 (0.05)	.82	1.14 (0.26)	.61
Postpolicy trend change (β_3_)^e^	1.03 (0.01)	<.001	1.17 (0.04)	<.001

^a^ Analysis used Kroger Pharmacy data.

^b^ Physician proxy designates the rate of naloxone orders dispensed by the physician who authorized the Kroger Pharmacy physician-approved protocol.

^c^ The coefficient β_1_ is the effect of the continuous time variable, beginning with a value of 1 on July 16, 2014, and increasing by 1 each month. This coefficient represents a monthly time series.

^d^ The coefficient β_2_ is the effect of a binary indicator representing policy implementation on July 16, 2015, with a value of 0 before July 16, 2015, and 1 after July 16, 2015. This coefficient represents the immediate change in rate of naloxone orders after policy implementation.

^e^ The coefficient β_3_ is the effect of a continuous variable measuring the time passed after the implementation of the policy, beginning with a value of 0 and increasing by 1 each month; non-0 values began on August 16, 2015, 1 month after the policy was implemented.

## Discussion

The implementation of an Ohio law allowing pharmacists to dispense naloxone in accordance with a physician-approved protocol was associated with an increase in the number of naloxone orders dispensed. These findings were evident in the Ohio Medicaid population and confirmed in the all-payer population. Our findings are consistent with those of a study by Xu et al,^18^ which found that the implementation of naloxone-related access laws, specifically standing-order and third-party prescribing provisions, was associated with a per-state increase of 48 in standing-order and 72 in third-party naloxone orders dispensed per quarter during a 10-year period. However, our results are in contrast to findings from a study by Abouk et al,^19^ which examined the association between state laws that facilitate pharmacy distribution of naloxone and the risk of fatal overdose. This study reported that there was little evidence of a trend change in the provision of Medicaid naloxone orders dispensed via indirect authority, which was defined as naloxone dispensed either through a standing order or a statewide protocol issued by a state health official for all licensed pharmacists.^19^ Although we do not know the reason for the disagreement in our findings, perhaps the aggregation of all states in the Abouk et al study did not allow for enough sensitivity to detect a difference in the trend.

In the Ohio Medicaid population, our study found a significant increase in the naloxone dispensing rate in counties with low employment. The rate of naloxone dispensed in low-employment counties increased by 18% in the postpolicy period. A recent study by the US Department of Health and Human Services reported that unemployment rates were highly correlated with the magnitude of the opioid crisis.^20^ A 3.8% increase in opioid per capita sales and a 4.6% increase in opioid overdose–related death rates with every 1% increase in a county’s unemployment rate was observed.^20^ Hollingsworth et al^21^ examined whether deaths and emergency department visits associated with the use of opioids and other drugs were associated with local unemployment rates and found that an increase in county unemployment rates was associated with an increase in opioid-related death rates. Our study expands on the previous studies by observing an association between naloxone dispensing rates and low-employment counties. Notably, our findings also suggest that the policy change was associated with increases in access to naloxone among patients residing in these vulnerable areas.

Many organizations have recognized opioid misuse as a public health challenge. The Office of the Surgeon General released a public health advisory in 2018, its first in 13 years, recommending that Americans carry naloxone.^22^ In 2017, the opioid crisis was declared a public health emergency by the president of the United States, and a 5-point strategy was outlined, which focused on improving access to prevention, treatment, and recovery services; targeting the availability and distribution of overdose-reversing drugs; strengthening public health data collection and reporting; supporting addiction and pain research; and advancing the practice of pain management.^23^ The US Food and Drug Administration is now considering allowing naloxone to be available over the counter, and 14 states are already in the process of doing so.^24,25^ Such reclassification of naloxone could lead to a considerable increase in naloxone use.^26^ Pharmacists are in a strong position to support these strategies because they serve as patient educators, provide recommendations for the appropriate use of opioids and their adverse effects, and can advise patients and their family members about the availability of naloxone.^27^ A study by Thompson et al^28^ evaluated pharmacists’ knowledge of naloxone and the Ohio law, perceived barriers that may prohibit the dispensing of naloxone, and Ohio pharmacists’ general confidence, comfort, perception, and experience with dispensing naloxone per a physician-approved protocol. The authors reported that most of the pharmacists were confident they possessed the knowledge and training necessary to identify patients at risk of an opioid overdose and did not perceive their knowledge and training as barriers to dispensing naloxone. Moreover, pharmacists are able to provide medication-assisted treatment and can work with health care teams to optimize pain treatment.^29^ Collaboration with physicians and, indeed, the entire health care team to provide overall care and prevention is important to address issues as complex as addiction.

Many consumers are not aware of the risks associated with opioid use or may be unfamiliar with increased access to naloxone, so they may not initiate conversations about naloxone with health care professionals and may miss the potential benefits of having naloxone available.^30^ Hence, part of the success of the implementation of a physician-approved protocol depends on a pharmacist’s ability to identify and educate patients who might benefit from the protocol.^31^ A study by Green et al^32^ discovered 2 common themes from consumer and pharmacist perspectives about naloxone: (1) consumers may be unaware of the need for naloxone for themselves or their loved ones and its availability at the pharmacy, and (2) both consumers and pharmacists may experience stigma and discomfort in asking for or offering naloxone. The study suggested that a universal offer should be made to provide naloxone at pharmacies, and pharmacists should offer naloxone as a part of a universal opt-out strategy to all patients who are perceived as having a high risk of opioid misuse.

### Limitations

Our study had several limitations. We did not measure whether a change in the rate of opioid misuse occurred in the postpolicy period nor did we include opioid-related mortality data; however, one of the major strengths of this study was its use of an interrupted time-series design, which adjusted for the consequences of the policy implementation by considering the change in naloxone orders dispensing that would have occurred in the absence of the policy.^16^ In addition, for the overall model, we assumed that all naloxone orders in the postpolicy period were dispensed in accordance with the physician-approved protocol because a method did not exist to distinguish them from personal naloxone prescriptions; however, we created a physician-proxy model to account for this limitation. We also used data from only 1 state; however, the misuse of opioids in Ohio is representative of issues experienced throughout the country.

## Conclusions

Opioid misuse is a complex issue that requires a multifaceted prevention and treatment approach. Laws intended to protect the public may hinder access to naloxone. The implementation of a new law in Ohio, which allowed pharmacists to provide naloxone without a prescription in accordance with a physician-approved protocol, was associated with increased naloxone dispensing rates, especially in low-education and high-poverty counties. Pharmacist provision of naloxone may improve access to this important medication.
