# Error in Conclusions

**DOI:** 10.1001/jamanetworkopen.2020.1568

**Published:** 2020-03-02

In the Original Investigation titled “Association Between a State Law Allowing Pharmacists to Dispense Naloxone Without a Prescription and Naloxone Dispensing Rates,”^1^ published January 31, 2020, there was an error in the third sentence of the Conclusions. The phrase given as “low-education” should have been “low-employment.” This article has been corrected.^1^
